# Diagnostic Value of the Sentinel Lymph Node Technique in Patients with Muscle-Invasive Bladder Cancer

**DOI:** 10.3390/jcm12093092

**Published:** 2023-04-24

**Authors:** Bartosz Małkiewicz, Diana Jędrzejuk, Adam Gurwin, Karol Wilk, Klaudia Knecht-Gurwin, Paweł Kiełb, Wojciech Krajewski, Marek Bolanowski, Agnieszka Hałoń, Tomasz Szydełko

**Affiliations:** 1 Department of Minimally Invasive and Robotic Urology, University Center of Excellence in Urology, Wroclaw Medical University, 50-556 Wrocław, Poland; 2 Department of Endocrinology, Diabetes and Isotope Therapy, Wroclaw Medical University, 50-367 Wrocław, Poland; 3 Department of Dermatology, Venereology and Allergology, Wroclaw Medical University, 50-368 Wroclaw, Poland; 4 Department of Clinical and Experimental Pathology, Wrocław Medical University, 50-556 Wrocław, Poland

**Keywords:** sentinel node, lymph node metastasis, lymphadenectomy, cystectomy, bladder cancer, SPECT/CT

## Abstract

Background: The optimal limits of the bilateral pelvic lymph node dissection (PLND) template in bladder cancer treatment remain controversial. This study aimed to investigate whether radio-guided sentinel node (SLN) detection is a reliable technique for the perioperative localisation of potential lymphatic metastasis during cystectomy for muscle-invasive bladder cancer (MIBC). Materials and Methods: We studied 54 patients with pT2-pT4 MIBC who underwent cystectomy with extended PLND (ePLND) augmented by the SLN technique. The identification of SLN was performed by preoperative SPECT/CT hybrid lymphoscintigraphy using peritumoral injection of nanocolloid-Tc-99m, followed by intraoperative navigation with a handheld γ-probe. All nodal specimens were collected separately and then fixed in formalin, stained with haematoxylin and eosin, and examined by an experienced uropathologist. Results: A total of 1414 LNs were resected and examined for the presence of metastases. The mean number of harvested LNs was 26 (range: 11–50) per patient. In 51 of 54 patients, 192 SLNs were resected. In addition, 20/192 (10.4%) SLNs were located outside of the ePLND area. Overall, 72 metastatic LNs (LN+) were found in 22 of 54 patients (40.7%) and in 24/192 SLNs (12.5%). The SLN technique detected LN+ in 14 of 22 (64%) patients. The SLNs were the only sites of metastasis (SLN+ = LN+) in 6 of 22 (27.3%) LN+ patients, including two cases with foci located in the pararectal region. The diagnostic values for the sensitivity, specificity, positive predictive value, and false-negative rate for the SLN technique were 66.66%, 4.16%, 28.57%, and 33.33%, respectively. Extended lymphadenectomy and its combination with the SLN technique enabled the correct assessment in 96.3 and 100% of patients, respectively. Conclusions: The combination of ePLND and SLN provides a better pN assessment compared to ePLND alone. Although the SLN technique has restrictions that limit its diagnostic value, its use as an addition to lymphadenectomy allows for the visualisation of nonstandard lymph drainage pathways that may be potential metastatic routes.

## 1. Introduction

The World Health Organization ranks bladder cancer as the ninth most common malignancy and the 13th most common cause of cancer-related death in the world [1,2]. Despite the increase in the global incidence of bladder cancer, a reduction in the mortality rate has been observed in recent years [3]. Overall, patients may be diagnosed with one of the two main histopathological stages: non-muscle invasive bladder cancer (NMIBC, stage pTa or pT1), which represents approximately 70% of cases, and tumour-invading detrusor muscle (muscle-invasive bladder cancer MIBC; stage T2–T4), constituting the remaining 30% of cases. Despite an optimal treatment and no histopathological findings of lymph node (LN) metastases, MIBC is associated with a 50% mortality rate over 5 years [4]. This indicates that half of patients diagnosed with MIBC have cancer metastases that are not detected by currently used staging techniques.

As the sensitivity of noninvasive diagnostic methods for detecting LN metastases, which are associated with negative prognosis, is inadequate, the gold standard surgical treatment of patients with MIBC is radical cystectomy with bilateral pelvic lymphadenectomy (PLND) [5,6,7]. Although PLND is known to have diagnostic and therapeutic value for bladder cancer, the optimal template limits remain controversial [8]. The sentinel lymph node (SLN) is the first LN or group of LNs on a direct lymphatic drainage pathway from the site of the primary tumour, which reflects the pathological status of the remaining lymphatic region, and acts as a “distribution station” for the spread of tumour cells to distal organs. The SLN mapping procedure helps to define the extent of lymphadenectomy to prevent blind and unnecessary treatment. The utilisation of this technique has been demonstrated to be useful in assessing the lymphatic spread of several tumour types, such as breast cancer, melanoma, penile cancer, and cervical cancer [9,10,11,12].

In the present study, we investigated the value of radio-guided SLN detection as a reliable technique for the perioperative localisation of potential metastatic roots and whether it can be utilised to assess the extent of PLND during cystectomy for MIBC.

## 2. Materials and Methods

### 2.1. Patients and Study Design

This was an interventional study based on the SLN concept. Between March 2015 and October 2018, 54 out of 165 patients with MIBC who were scheduled for radical cystectomy with extended PLND (ePLND) met the inclusion criteria of the study and consented to the administration of radiocolloid followed by hybrid SPECT-CT lymphoscintigraphy. The inclusion criteria were (1) pathological stage pT ≥ 2; (2) absence of pelvic LN metastases on contrast-enhanced CT or MRI (i.e., ≥8 mm in the short axis); (3) no evidence of distant metastasis on contrast-enhanced CT or MRI; (4) World Health Organization performance status of 0 or 1; and (5) no preoperatively known factors that may affect lymphatic drainage. Preoperative systemic chemotherapy was allowed. In all cases, surgery was performed with curative intent. The primary tumour was staged by conventional cross-sectional imaging. The study was conducted in accordance with the Declaration of Helsinki and approved by the Regional Ethics Committee. Written informed consent was obtained from all patients.

### 2.2. Radiocolloid Injection and SPECT/CT Imaging

The Tc-99m nanocolloid (Nanocoll; GE Healthcare, Milano, Italy) injections were performed on the day before planned surgery under local anaesthesia during cystoscopy. There were four radiotracer injections of 50 MBq with a volume of 1 mL each into the detrusor muscle around the tumour borders using a 3.7Fr Williams cystoscopic needle (Cook Urological, Spencer, IN, USA). For more than one lesion in the bladder, the injections were located around the largest focus. After bladder emptying, the radioactivity from the pelvic region was measured to assess the correct application of the marker and deposit estimation prior to lymphatic distribution.

**Figure 1:**
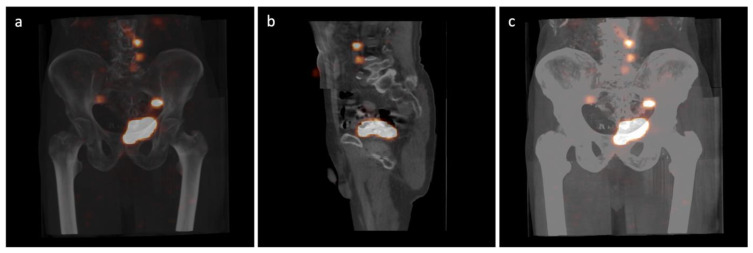
Sentinel lymph node (SLN) procedure in a bladder cancer patient. Fused SPECT-CT images to facilitate the anatomical identification of the SLNs: (**a**) frontal exposition; (**b**) sagittal exposition; (**c**) skeletal filtering.

Approximately 2 to 3 h after radionuclide injection, hybrid SPECT-CT lymphoscintigraphy was performed on a Gamma Camera BrightView XCT (Philips Healthcare, San Jose, CA, USA) equipped with low-energy, high-resolution collimators (LEHR). The low-dose CT projection was carried out using the noncontrast option. The study was conducted for approximately 30 min. The following image acquisition parameters were used: matrix size of 64 × 64, 64 strokes for each head, counting time of 20 s per image and 3 fields, 1 mm layer, 512 × 512 matrix size, 120 keV voltage and 20 mA current for the standard and iterative reconstructions, and 120 keV voltage and 20 mA current for the SPECT and XCT projections, respectively. The SOFT TISSUE filter (0.6) and the option for breathing correction were used. In order to facilitate the intraoperative anatomical location of active foci, the images obtained from SPECT and CT were fused and reconstructed using Extended Brilliance Workspace V1.0 software (Philips Medical Systems Nederland B.V.). Radionuclide uptake sites whose activity was significantly higher than the background and topographically unrelated to the injection site, rectum, bone marrow, kidneys, or liver were considered draining lymph nodes (SLNs). The reconstructed images were used for intraoperative navigation and the location of SLNs (Figure 1).

### 2.3. Surgical Procedure

**Figure 2:**
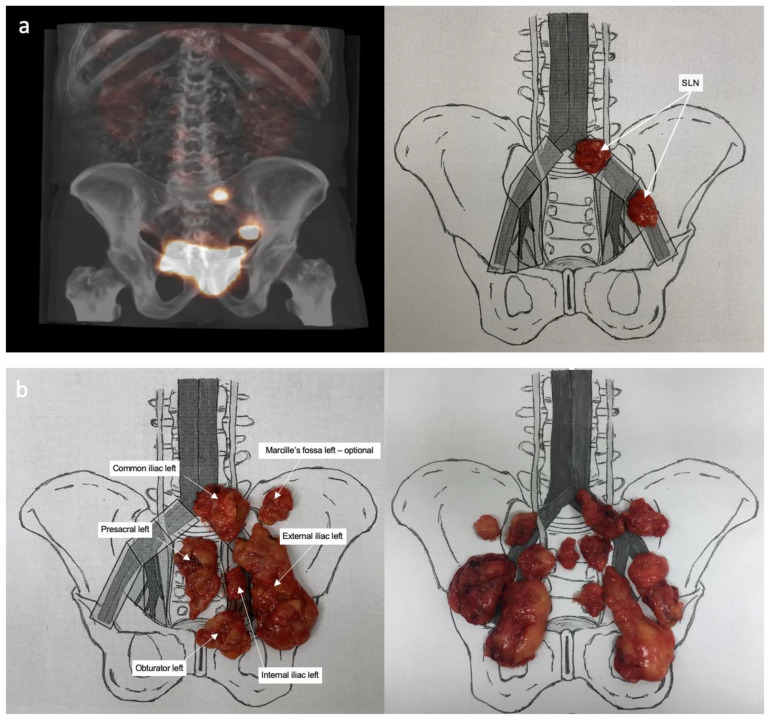
(**a**) Sentinel lymph nodes (SLNs) localized using SPECT-CT images and mapped on the template; (**b**) lymphadenectomy specimens corresponding to specific anatomical stations.

Radical cystectomy and ePLND were performed in all patients using the open technique. Under the guidance of the images from the SPECT/CT fusion, all lymph drainage stations were scanned methodically using the handheld gamma radiation probe (FlexProbe CCXS-OP-FP, CrystalPhotonics Gmbh, Berlin, Germany). To enable accurate orientation and minimise the risk of artefacts, a probe with a collimator and angled tip was utilised. A constant, intensive radioactivity reading that was at least ten times the background measurement was determined as a positive signal. After locating the SLNs, tissues were selectively removed and mapped on a diagram (Figure 2a). Then, ePLND was systematically performed within defined anatomical landmarks. The cranial limit of the LN dissection was the aortic bifurcation of the common iliac vein immediately superior to the confluence of the external and internal iliac veins. The caudal extension of the LN dissection was at the level of Cooper’s ligament, with the genitofemoral nerve as the lateral boundary. On both sides of the pelvis, lymphatic and fibro-fatty tissues were removed separately from the following anatomical stations: obturator fossa; external iliac; internal iliac; presacral; Marcille’s fossa; and common iliac. The location of the SLN outside the lymphadenectomy template was measured using the SPECT/CT images, and the activity was also confirmed by an intraoperative probe. In such cases, only active tissues were removed for examination without extending the resection area, to minimize the risk of complications. It concerned locations above the aortic and inferior cava vein bifurcations. Similarly, pararectal nodes located below the pararectal fascia close to the rectal muscular layer were removed only when a radioactivity signal was present. Finally, lymph and fibrous fatty tissues harvested from each anatomical region of the lymphadenectomy were screened ex vivo for the presence of radioactivity that could be missed at the first exposure and then mapped on a chart (Figure 2b).

### 2.4. Histopathological Evaluation

The cystectomy specimens were graded according to the WHO 1999 and WHO 2004 systems, as well as the TNM 2010 classification system [13]. All lymphatic tissues were submitted for histopathological examination as separate samples. The examination was performed by an experienced uropathologist. After fixation, the specimens were palpated, visually inspected, sectioned, stained with haematoxylin and eosin, and embedded in paraffin. The samples were subjected to two identical rounds of analysis to reduce the risk of lab error. The LNs were counted and microscopically assessed for the presence of micro- and macro-metastases (<0.2 mm and >0.2 mm, respectively) of extracapsular infiltration and cancer embolus in the lymph vessels. In the case of the presence of malignant cells in the perivesical adipose tissue without the presence of anatomical structures typical for the LN (capsule and subcapsular sinus), the N+ feature was not stated.

### 2.5. Definitions and Statistical Analysis

The binary and discrete data are presented as counts and percentages in cross-reference tables. Continuous variables are presented as means and standard deviations (SDs) or medians and interquartile ranges (IQRs). The verification of the hypothesis concerning the equality of the average parameters in the dependent groups was assessed using the nonparametric Wilcoxon signed-ranks test. Ninety-five percent confidence intervals were determined.

An SLN was defined as an LN expressing radioactivity at each anatomical LN station. The concordance between the SLN pathological evaluation and the definitive pathological status of LNs in the same anatomical site was calculated by the sensitivity, specificity, accuracy, false-negative rate, and positive and negative predictive values. The test results were defined as follows: true positive (TP)—SLNs detected, and metastasis found in at least one SLN; false positive (FP)—SLNs detected but no metastases in the SLNs; true negative (TN)—no SLNs detected and no metastases in the SLNs and other LNs; and false negative (FN)—SLNs detected but metastases found only in nodes other than the SLNs. The individual parameters of the diagnostic test were calculated based on the following formulas: sensitivity = (TP/(TP + FN)) × 100; specificity = (TN/(FP + TN)) × 100; positive predictive value, PPV = (TP/(TP + FP)) × 100; negative predictive value, NPV = (TN/(TN + FN)) × 100; accuracy, ACC = ((TP + TN)/TP + FP + TN + FN) × 100; and FN rate = (FN/(TP + FN)) × 100. For a better description of the diagnostic value, the relative risk (RR) and likelihood ratios (LRs) were calculated. We assumed that the pathological examination used as the reference method provided 100% certainty in determining the presence of metastases and that both the SLN detection methods using a γ-probe and SPECT/CT have undetermined diagnostic values. All statistical analyses were performed using Statistica v.13.3 (TIBCO Software Inc., Palo Alto, CA, USA).

## 3. Results

### 3.1. Patients and Disease Characteristics

Of the 54 patients included in the study, 43 (79.6%) were male. The median age of the patients was 66.9 years (min–max: 44–82). All patients had negative surgical margins and 36 (66.7%) non-organ-confined tumours (stage T3 or T4). Neoadjuvant systemic chemotherapy was administered in 24 (44.4%) cases. A total of 1414 LNs were resected and examined for the presence of metastases. The mean number of harvested LNs was 26 (range: 11–50) per patient. There were 32 (59.3%) patients free of LN metastasis. Table 1 depicts the basic patient and disease characteristics.

### 3.2. Sentinel Lymph Node Detection

Preoperative SPECT-CT imaging and intraoperative γ-probe scanning were applied to all patients. In 51 of 54 patients (94.4%), the number of hot-spot foci was consistent between the SPECT-CT and γ-probe imaging. In the remaining three patients, no SLNs were detected with either method. To confirm the homogeneity of both techniques, two statistical tests were performed to assess compliance: McNemar’s test (*p* = 0.929) and Wilcoxon matched-pairs signed-ranks test (*p* = 1.00). An average of 3.57 SLNs per patient were identified using SPECT-CT. An identical result indicated the intraoperative location with the γ-probe.

### 3.3. Lymph Node Characteristics

**Figure 3:**
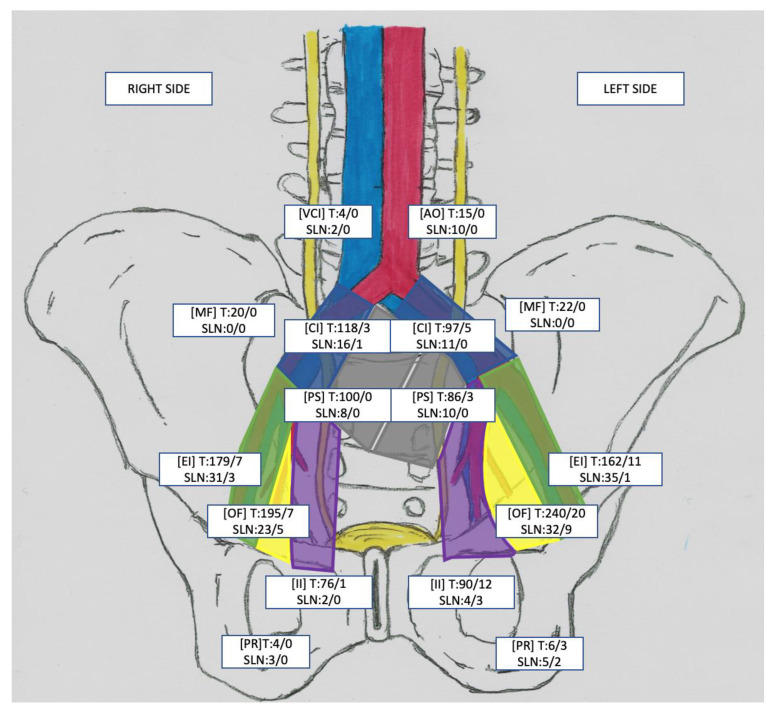
Location and number of dissected lymph node (LN) stations, pathologically positive stations, sentinel lymph nodes (SLNs), and pathologically positive SLNs. Each square represents the anatomical LN stations of the EI (external iliac), OF (obturator fossa), CI (common iliac), PS (presacral), PR (pararectal), II (internal iliac), MF (Marcille’s fossa), and VCI (paracaval) or AO (paraaortic) on the right and left sides of the pelvis, respectively. The figures in each square represent the following: first line—T, number of resected LNs/number of positive LNs (LN+); second line—SLNs detected in the anatomical station/positive SLNs (SLN+) in the anatomical station.

In total, 192 SLNs were resected from 54 patients (median: 6; interquartile range (IQR): 3–9; including three patients without the presence of an SLN). In 31/54 (57.4%) cases, SLNs were located unilaterally. The template of the ePLND did not cover all SLNs in 10/51 (19.6%) patients, which resulted in a total of 20/192 (10.4%) SLNs outside of the ePLND template. Out of 192 SLNs, 24 (12.5%) were metastatic nodes (SLN+). Overall, 72 metastatic LNs (LN+) were found in 22 of 54 patients (40.7%). SLN+ accounted for 33% (24/72) of all LN+ removed. The use of the SLN technique alone would allow for the detection of LN+ disease in 14/22 (64%) patients. In only 6/22 (27.3%) of the LN+ patients, the SLNs were the only sites of metastasis (SLN+ = LN+). In two of them (2/22; 9.1%), the SLN+ was outside of the ePLND template (both patients with one SLN+ in the pararectal region). In 8/22 (36.4%) LN+ patients, metastases in 29 LNs were found in other locations that did not show uptake of the radiotracer and were detected only by performing ePLND. A detailed topography of the SLNs and the number of dissected LN stations are presented in Figure 3. Moreover, in Table 2 a comparison of the ePLND and SLN performances is provided.

### 3.4. SLN Technique’s Diagnostic Values

The obtained pathological data and the results of the SLN detection enabled the calculation of the diagnostic parameters of the SLN technique for the SPECT/CT and intraoperative γ-probe. In the studied cohort, the following structures of the γ-probe and SPECT/CT validity parameters were demonstrated: TP—14, FP—30, TN—2, and FN—8 and TP—14, FP—30, TN—3, and FN—7. The diagnostic values for the sensitivity, specificity, positive predictive value, and false-negative rate for the SLN γ-probe technique were 63.64%, 6.25%, 31.82%, and 36.36%, respectively. The remaining diagnostic parameters of the γ-probe and SPECT/CT are presented in Table 3.

The difference observed between the two tested methods was very small: in one patient, the γ-probe method showed one SLN more than the SPECT/CT, and in the other, the SPECT/CT showed one SLN more than the γ-probe method.

### 3.5. Morbidity

Postoperative morbidity connected with lymphadenectomy was recognised in 23 out of 54 patients (42.6%). Among the complications associated directly with ePLND, there was lymphorrhea, which occurred in 21 patients, and symptomatic lymphocele in two patients. There were no complications specific to the SLN technique.

## 4. Discussion

The underlying idea behind preoperative or intraoperative SLN dissection is the assumption that negative SLNs reflect a regional group of tumour-free LNs. For this reason, PLND can be avoided entirely, or the lymphadenectomy template can be altered when the SLN detected is free of metastases [9]. SLN biopsy in patients with MIBC was first introduced in 2001 by Sherif et al. Only a few other groups have studied this concept and demonstrated the importance of SLNs as a general indicator of pelvic LN involvement, which can improve the assessment of pN staging by identifying metastases in patients without clinical evidence of nodal metastases [14,15,16].

Our study demonstrated a detection rate of 94.5% (51 out of 54 cases) for SLNs using a radiotracer, confirming previous reports that intraoperative SLN mapping is a feasible procedure in MIBC patients [17]. The feasibility of sentinel node biopsy in MIBC has been investigated using various techniques, including preoperative and intraoperative detection methods using radiotracers [18]. A recent meta-analysis in this area showed a 91% detection rate (95% CI: 87–93%) and a 79% sensitivity (95% CI: 0.69–0.86%), aggregating data from eight publications [19]. The high detection rate of SLNs in MIBC, consistent with our results, indicates a realistic possibility of detecting SLN intraoperatively using a gamma probe. Although previous studies have used other methods, including blue dye and preoperative lymphoscintigraphy, intraoperative mapping of SLNs using a handheld gamma probe has demonstrated a higher detection rate than the methods mentioned above. Our results are consistent with previous studies on SLN mapping in other malignancies, such as breast cancer [20,21].

As in the current analysis, the authors located more than one SLN in other studies evaluating the SLN technique in MIBC [16,17,22]. In our study, metastases in SLNs were found in 14 of 54 patients (26%), constituting almost 64% (*n* = 22) of LN+ patients. In 6 of 14 (43%) patients with involved SLNs, these were the only sites of metastases, i.e., hot-spot foci were also metastatic nodes (SLN = LN+). Given the topography of metastatic SLNs, the predominant number was again located within the standard area of the ePLND (21/24). Only one metastatic hot spot was within the common iliac nodes. Interestingly, three patients had metastatic SLNs outside of the ePLND template. In one patient, the metastatic hot spot was found within the common iliac nodes. The other two patients had metastatic SLNs detected in the perirectal area. To the best of our knowledge, metastasis to the perirectal nodes has not been reported separately in the literature for lymphadenectomy in bladder cancer, and this is the first report.

Like other midline structures, the bladder has a bilateral lymphatic flow, anatomically different lymphatic drainage in patients, and a highly variable location of sentinel nodes [23]. The bilateral distribution of lymphatic drainage, independent of the tumour side, is called the crossover phenomenon. It can lead to the coexistence of SLNs from different lymphatic pathways and the occurrence of contralateral metastases. Therefore, defining the first SLN would be mainly of academic significance, and the concept of a single tumour node must be questioned concerning MIBC. Based on available studies, it is unknown whether the distinction between first-, second-, and third-order sentinel nodes is vital in this type of cancer [24,25,26]. In addition, it is doubtful that a general pattern of lymph flow typical to most patients can be established depending on the size and location of the primary tumour [27]. In MIBC, as in other midline cancers, such as penile, uterine, vulvar, and anal cancers, lateralisation of sentinel node distribution has been observed, which is also the case in our study [17,28,29,30]. On the other hand, the bilateral distribution of SLNs in MIBC has also been reported [16,31].

The most important single diagnostic parameter for the SLN technique is the false-negative score rate (FNR), i.e., cases in which there are no hot spots, or they are in other places than the actual nodal metastases. In our study, we obtained an FNR of 33.33%. This is the highest value among all papers studying the significance of SLNs in MIBC. Aljabery et al. determined false-negative results at the level of 19%. However, this calculation was defined as the percentage of patients with detected nonmetastatic sentinel nodes with metastases found in other nodes [16]. Połom et al. determined the FNR to be 8%, using the same methodology but with a smaller number of cases [22]. The most proportionally similar result to that obtained in the presented analysis was obtained by Liedberg et al., who found a false-negative SLN in 6 of 32 LN+ patients, and the FNR was calculated at 19% [17]. The authors suggested that the pathological SLNs were obstructed by tumour cells, resulting in a lack of radiolabel uptake on one side of the pelvis and excessive drainage of LNs on the opposite side. Based on other malignancies, it has been shown that a high tumour T stage is paradoxically associated with a higher failure rate of SLN detection due to the fact of lymphatic vessel compression and infiltration [32,33].

Our results demonstrate that most of the SLNs were localised in the external iliac vascular region, followed by the internal iliac fossa, antero-sacral region, obturator fossa, and division of the common iliac artery. Such a distribution of SLNs is consistent with previous results by other researchers [14,17,31]. The demonstration of alternative lymph drainage routes from the bladder prompted us to analyse the use of the SLN technique as an adjunct to lymphadenectomy. The ePLND applied to the patients allowed for a correct diagnosis of LN+ in 20 of 22 patients (90.9%) and the excision of 94.4% of nodal metastases. The complementation of the last range of SLN lymphadenectomy allowed for proper staging and the complete removal of the affected nodes in all patients from the LN+ group. According to our results, the detection of an SLN leads to better pelvic LN staging and avoids lymphadenectomy in patients without SLN metastases. However, if SLN detection fails, lymphadenectomy may still be necessary.

We acknowledge certain limitations of our study. Firstly, the study population was relatively small, and all enrolled patients were admitted to one tertiary centre. The small number of patients was due to the fact of our concerns associated with significantly extended LND and possible complications. Secondly, not all patients included in our study received neoadjuvant chemotherapy (NAC). Nevertheless, as Rosenblatt et al. have demonstrated, it is not a mandatory condition [15]. In some cancers, such as oesophageal cancer, past NAC reduces the frequency of the detection and accuracy of sentinel node mapping, but no such relationship has been reported in MIBC [34]. Moreover, intraoperative SLN biopsy may be suggested as a feasible method for patients with MIBC after NAC.

Given the results obtained and the limited data from the world literature, it should be concluded that the SLN technique cannot be a surrogate for lymphadenectomy for the time being. Low diagnostic parameters and the underestimation of the staging of the pN feature at the level of 30% are unacceptable [16]. At the same time, current observations indicate that the SLN technique should be a complement to lymphadenectomy and not the only method performed for LN evaluation. It can show nonstandard lymph drainage channels, which in some patients may be the only route of metastasis. On this basis, a complete rejection of the therapeutic impact of the SLN technique seems unjustified but undoubtedly requires further research.

## 5. Conclusions

SLN biopsy is a feasible method for the perioperative assessment of LN status in MIBC, but the effectiveness of this technique in MIBC as a standalone method is limited. Nevertheless, it enables the visualisation of nonstandard lymphatic drainage pathways that may be potential metastasis routes, thereby improving ePLND outcomes. The application of the SLN technique in MIBC has not yet been elucidated and requires further research.

## Figures and Tables

**Table 1:** Baseline patient and disease characteristics.

Variable	Total N = 54	
**Age** (years):		
Mean ± SD	66.9 ± 8.0	
Median (Q1, Q3)	68 (61, 72)	
Min–Max	44–82	
**Gender:**	*n*	%
Male	43	79.6
Female	11	20.4
**ACCI:**		
Mean ± SD	6.6 ± 1.7	
Median (Q1, Q3)	7 (5, 8)	
Min–Max	3–10	
**BMI** (kg/m^2^):		
Mean ± SD	26.0 ± 4.7	
Median (Q1, Q3)	26 (23, 28)	
Min–Max	17–43	
**Neoadjuvant chemotherapy:**		
Yes	24	44.4
No	30	55.6
**pT:**	*n*	%
pT2	18	33.3
pT3	19	35.2
pT4	17	31.5
**pN:**	*n*	%
pN0	32	59.3
pN1	7	13.0
pN > 1	15	27.8
**Number of lymph nodes removed (LNs):**		
Total Mean ± SD	1414 26.2 ± 9.2	
Median (Q1, Q3)	23 (20, 32)	
Min–Max	11–50	
**Number of lymph nodes involved (LN+):**		
Mean ± SD	1.3 ± 2.8	
Median (Q1, Q3)	0 (0, 2)	
Min–Max	0–14	

**Table 2:** Comparison of the extended pelvic lymph node dissection (ePLND) and sentinel lymph node (SLN) dissection performances.

Scope of LN Dissection	LN+ Patients with Correct Staging (%, 95% CI)	LN+ Patients with All Positive LNs Removed (%, 95% CI)	LN+ Removed (%, 95% CI)	LNs That Need to Be Removed (%, 95% CI)
ePLND	20/22 90.9 (78.9–100.0)	19/22 86.4 (72.0–100.0)	68/72 94.4 (89.2–99.7	1385/1414 97.9 (97.2–98.7)
SLN	14/22 63.6 (43.6–83.7)	6/22 27.3 (8.7–45.9)	24/72 33.3 (22.4–44.2)	192/1414 13.6 (11.8–15.4)
ePLND + SLN	22/22 100.0 (100–100)	22/22 100.0 (100–100)	72/72 100.0 (100–100)	1414/1414 100 (100–100)

**Table 3:** Diagnostic values of the sentinel lymph node technique.

Diagnostic Parameter *	γ-Probe	SPECT/CT
Sensitivity	63.64%	66.67%
Specificity	6.25%	9.09%
Positive predictive value (PPV)	31.82%	31.82%
Negative predictive value (NPV)	20.00%	30.00%
Accuracy (ACC)	29.63%	31.48%
False-negative rate	36.36%	33.33%
Relative risk (RR)	39.77%	45.45%
Likelihood ratio + (LR+)	0.68	0.73
Likelihood ratio − (LR−)	5.82	3.67

* Validity parameters of the diagnostic test calculated based on the multiplicity: γ-probe (PD = 14, FD = 30, PN = 2, and FN = 8) and SPECT-CT (PD = 14, FD = 30, PN = 3, and FN = 7).

## Data Availability

The data are available from the authors upon reasonable request.
